# Kazakhstan can achieve ambitious HIV targets despite expected donor withdrawal by combining improved ART procurement mechanisms with allocative and implementation efficiencies

**DOI:** 10.1371/journal.pone.0169530

**Published:** 2017-02-16

**Authors:** Andrew J. Shattock, Clemens Benedikt, Aliya Bokazhanova, Predrag Đurić, Irina Petrenko, Lolita Ganina, Sherrie L. Kelly, Robyn M. Stuart, Cliff C. Kerr, Tatiana Vinichenko, Shufang Zhang, Christoph Hamelmann, Manoela Manova, Emiko Masaki, David P. Wilson, Richard T. Gray

**Affiliations:** 1 The Kirby Institute, University of New South Wales, Sydney, Australia; 2 The World Bank Group, Washington DC, United States of America; 3 Joint United Nations Programme on HIV/AIDS, Geneva, Switzerland; 4 United Nations Development Programme, Istanbul Regional Hub, Istanbul, Turkey; 5 Republican Center for Prevention and Control of AIDS, Almaty, Kazakhstan; 6 The Burnet Institute, Melbourne, Australia; 7 Department of Mathematical Sciences, University of Copenhagen, Copenhagen, Denmark; 8 School of Physics, University of Sydney, Sydney, Australia; 9 The Global Fund to Fight AIDS, Tuberculosis and Malaria, Geneva, Switzerland; UCL, UNITED KINGDOM

## Abstract

**Background:**

Despite a non-decreasing HIV epidemic, international donors are soon expected to withdraw funding from Kazakhstan. Here we analyze how allocative, implementation, and technical efficiencies could strengthen the national HIV response under assumptions of future budget levels.

**Methodology:**

We used the Optima model to project future scenarios of the HIV epidemic in Kazakhstan that varied in future antiretroviral treatment unit costs and management expenditure—two areas identified for potential cost-reductions. We determined optimal allocations across HIV programs to satisfy either national targets or ambitious targets. For each scenario, we considered two cases of future HIV financing: the 2014 national budget maintained into the future and the 2014 budget without current international investment.

**Findings:**

Kazakhstan can achieve its national HIV targets with the current budget by (1) optimally re-allocating resources across programs and (2) either securing a 35% [30%–39%] reduction in antiretroviral treatment drug costs or reducing management costs by 44% [36%–58%] of 2014 levels. Alternatively, a combination of antiretroviral treatment and management cost-reductions could be sufficient. Furthermore, Kazakhstan can achieve ambitious targets of halving new infections and AIDS-related deaths by 2020 compared to 2014 levels by attaining a 67% reduction in antiretroviral treatment costs, a 19% [14%–27%] reduction in management costs, and allocating resources optimally.

**Significance:**

With Kazakhstan facing impending donor withdrawal, it is important for the HIV response to achieve more with available resources. This analysis can help to guide HIV response planners in directing available funding to achieve the greatest yield from investments. The key changes recommended were considered realistic by Kazakhstan country representatives.

## Introduction

Many countries are experiencing downward trends in their HIV epidemics after 10–15 years of large global investment [1–4]. However, some countries now face international donor withdrawal despite non-decreasing epidemics [5]. Kazakhstan has recently experienced a decrease in HIV funding from international donors after achieving upper-middle-income country status in 2006 [6]. In future, international aid for HIV is likely to be completely withdrawn for Kazakhstan. Although the government has increased domestic funding for the national response [6], in 2012 20% of spending still came from international donors [7].

**Fig 1:**
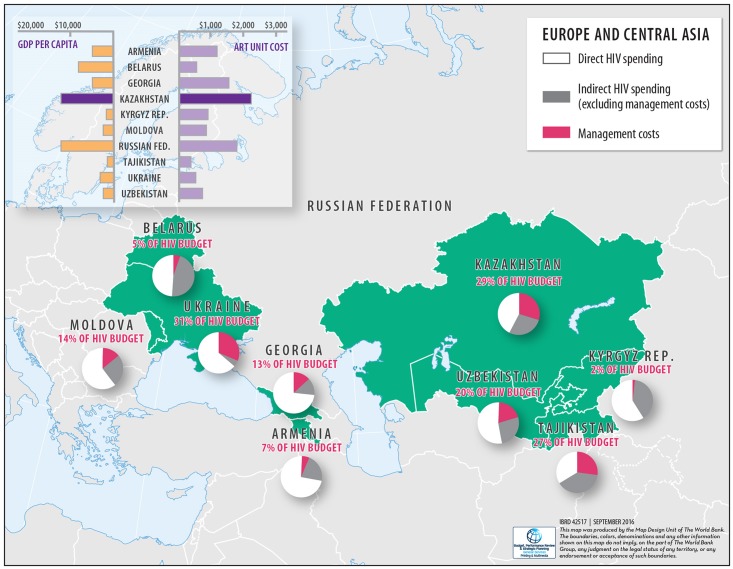
Gross domestic product per capita, ART unit costs, and key HIV/AIDS program spending data in selected EECA countries. This figure highlights key HIV-related spending data from selected countries in the Eastern Europe and Central Asia region. Only countries for which data were available are illustrated. The pie charts represent total HIV spending in 2014, and the bar graphs represent national gross domestic product (GDP) per capita and ART unit costs. The red text within the parentheses represents the proportion of the respective national budget consumed by management costs.

Currently Kazakhstan’s gross domestic product (GDP) per capita exceeds $12,000 USD. This surpasses many countries in the Eastern Europe and Central Asia (EECA) region, and suggests Kazakhstan is in a stronger position to fund its HIV response (Fig 1). However, this economic advantage is diminished by costs of procuring antiretroviral drugs (ART) being greater than other countries in the region, exceeding the EECA average (of countries for which recent data are available) by 110% (Fig 1). This high cost of HIV treatment is primarily driven by the exclusion of the Kazakhstan government from voluntary licence agreements that pharmaceutical companies negotiate with generic drug manufacturers due to Kazakhstan’s upper-middle-income country status [8]. Central Asia–along with Europe–experiences the highest cost of donor-funded ART when procured from originator companies, but also the least expensive when procured from generic manufacturers [9], highlighting the importance for countries in these regions to have access to the generic ART market. Such high treatment costs in the future would likely hinder the attainment of Kazakhstan’s HIV targets—especially after donor withdrawal. However, it is believed that future costs of ART can be substantially reduced through the more efficient pooled procurement processes of the United Nations and the Global Fund to Fight AIDS, Tuberculosis and Malaria (GFATM) [10, 11].

Like other former Soviet Union republics, Kazakhstan had an exploding HIV epidemic amongst people who inject drugs during the 1990s [12–14]. This epidemic was driven by a high proportion of adults living in towns along major drug trafficking routes injecting drugs (up to 10% of the population) [15]. Since 2000, the epidemic has accelerated, with new HIV infections increasingly reported amongst heterosexual partners of people who inject drugs, female sex workers and their clients, men who have sex with men, prison inmates, and migrants [13, 16]. By 2011, heterosexual infections had surpassed injecting drug use as the primary mode of HIV transmission [17, 18]. There were an estimated 20,000 people living with HIV in Kazakhstan in 2013 [19]. As of 2013, the estimated HIV prevalence among people who inject drugs was 8%, among prison inmates 3%, female sex workers 1.5%, and men who have sex with men 1.2% [20]. The priorities of the national strategic plan [21] are to stabilize the epidemic by increasing public awareness of HIV prevention, targeting key populations at higher risk of transmission, continuing to provide access to treatment, and virtually eliminating mother-to-child transmission [21, 22].

Within an HIV response, funding is required for critical enablers to support treatment and prevention programs. These “indirect” costs include management, coordination, enabling environment, training, and capacity building expenditures. In Kazakhstan, these indirect costs consumed 58% of the 2014 HIV budget, with 29% of the overall budget allocated to management costs. This proportional allocation to management programs in Kazakhstan is much higher than many countries in the EECA region (Fig 1). Similar findings have previously been reported by Forsythe et al [23]. Whilst this could partially be due to potential differences in accounting definitions, with staff and infrastructure costs either categorized within indirect or program costs, there is likely to exist opportunities to reduce management costs in Kazakhstan through implementation efficiencies. Obtaining efficiency gains in essential management programs would provide extra funds for prevention and treatment, allowing Kazakhstan to achieve more with its current resources.

Here we used mathematical modeling to explore the potential impact of three types of efficiency gains with respect to Kazakhstan’s national targets. The impact of funding the most cost-effective programs to an optimal level (known as allocative efficiency), decreasing the unit cost of treatment programs (technical efficiency), and increasing the productivity of indirect programs and overhead costs (implementation efficiency) [24] are simultaneously considered. We further investigated if ambitious targets could be achieved with such efficiencies.

## Methods

We conducted an allocative efficiency analysis using the Optima HIV model [25]. We modeled the interactions, HIV transmissions, and transitions within the general population and key populations using 12 distinct population groups: the general population segregated by sex and age (0–14 years, 15–49 years, and 50+ years), female sex workers and their clients, people who inject drugs (segregated by sex), men who have sex with men, and prison inmates. Demographic, behavioral, and biological data required to inform the model were collected from national vital statistics [19, 26], integrated bio-behavioral surveillance [17] and routine program records. Model projections based on these inputs were calibrated to align the model output with empirical epidemiological data collected from integrated bio-behavioral surveillance [17] and data from registers of HIV testing and counseling services. Details of the model calibration process are presented in the Supplementary Material (S1 and S2 Figs). The data used to inform the model were primarily collected by Kazakhstan representatives attending an allocative efficiency workshop held in November 2014 in Yerevan, Armenia.

Nine “direct” HIV prevention and treatment programs were modeled by defining relationships between program spending and relevant transmission-related outcomes. The programs modeled were female sex worker and client prevention programs, men who have sex with men prevention programs, programs for people who inject drugs, needle-syringe programs, opiate substitution therapy (OST), mass media programs, HIV testing and counseling programs, prevention of mother-to-child-transmission (PMTCT), and antiretroviral therapy (ART). Along with the aforementioned behavioral and epidemiological data, these spending-versus-outcome relationships were informed by unpublished annual HIV/AIDS national budgets and program expenditure data. We present the full set of relationships with associated uncertainties in S3 Fig.

As described in Kazakhstan’s national strategic plan, the priorities of the national HIV/AIDS response are to contain the epidemic within key populations, to restrict overall population prevalence to 0.2%-0.6%, and to prevent mother-to-child transmission (MTCT). We translated these goals into quantified targets within the Optima model. The Kazakhstan allocative efficiency study group interpreted the epidemic targets to mean no further increases in annual newly-acquired sexual and injecting HIV infections and no further increases in annual AIDS-related deaths by 2020 compared to 2014 levels. Prevention of MTCT is assumed to mean virtual elimination of mother-to-child-transmission as defined by UNAIDS [27, 28]. Ambitious targets were further defined as 50% reductions in annual new sexual- and injecting-related transmissions, a 50% reduction in annual AIDS-related deaths, and virtual elimination of mother-to-child transmission, all by 2020.

The annual HIV budget required for Kazakhstan to achieve their national targets should 2014 allocations be scaled up proportionally was determined. This was achieved by incrementally increasing the allocation to each program proportionally (and hence varying associated model inputs as defined by the cost-outcome relationships) and simulating the model to assess whether the epidemiological outcomes satisfied the targets. Both the additional cost and epidemiological gain of this increase in spending were compared to a ‘status-quo’ scenario of continuing 2014 spending policies for 6 years from 2015–2020.

A series of scenarios were then simulated to determine whether Kazakhstan could achieve their national targets—or further, the ambitious targets—by 2020 under assumptions of future efficiency gains and total HIV budget. For each scenario, three types of efficiency gains were considered: 1) gains in allocative efficiency through optimally redistributing direct funds, 2) gains in implementation efficiency through reducing management costs (with surplus funds re-allocated to direct programs), and 3) gains in technical efficiency through reducing ART procurement costs. Technical efficiencies of prevention programs were not considered. Through discussions with country representatives, reductions in future ART costs of up to 60%-70% were identified to be plausible, as were 20%-25% reductions in management costs. In order to fully scope the space of potential cost reductions, we considered (in percentage point increments) reductions in ART costs from 0% to 80%, and reductions in management costs from 0% to 50%. A scenario was simulated for each pairwise combination of ART cost reduction and management cost reduction, with the budget for direct programs in each scenario being optimized through a formal mathematical optimization algorithm [29] (which uses the cost-outcome relationships) representing maximal allocative efficiency. Two levels of future HIV financing were considered for each simulated scenario. The future annual HIV budget available was restricted to either a) 2014 levels of funding (where international funds would be replenished from domestic sources), or b) 2014 levels without international donor funding. In the second case, we assume that the 20% of the total budget currently sourced from international donors [7] is withdrawn proportionally from direct and indirect programs.

By simulating scenarios for each combination of implementation efficiency gain and ART cost reduction assumption, we were able to identify—for both assumptions of future total HIV budget—the scenarios for which the national and ambitious targets were attained when direct funds were optimally allocated. We then identified one key scenario that achieved a desirable outcome (that is, achievement of national or ambitious targets) whilst still being considered realistic in terms of the efficiency gains required. By comparison with status-quo, this key scenario was analyzed in terms of newly-acquired HIV infections averted and AIDS-related deaths averted.

## Results

In the absence of optimal allocative, implementation, and technical efficiencies a 32% [27%-42%] increase in HIV budget would be required to achieve Kazakhstan’s national targets if the current allocation to direct programs is scaled up proportionally. Whilst an estimated 4,090 newly-acquired HIV infections and 2,950 AIDS-related deaths would be averted by 2020 compared to status-quo spending, an additional $75.1 million [$63.7m-$96.38m] would be required, assuming indirect costs remain fixed at $21.8 million a year.

**Fig 2:**
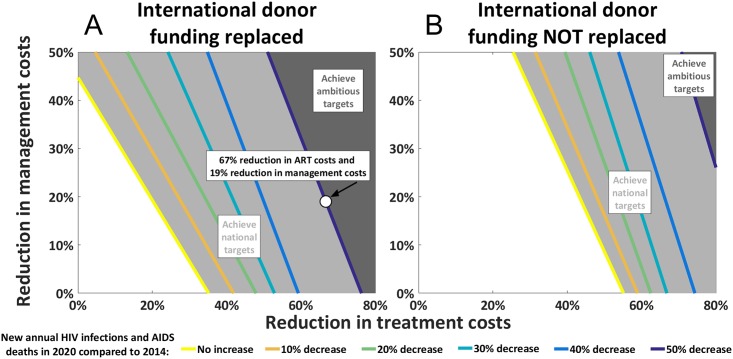
Contour plot of thresholds to achieve national and ambitious targets with varying levels of management cost reductions and treatment cost reductions. This figure illustrates the estimated reduction in management costs and treatment costs required to achieve i) national targets (light grey region), and ii) ambitious targets (dark grey region) should the annual budget be restricted to a) 2014 levels (Fig 2A), or b) 2014 levels without international donor funding (Fig 2B). The colored contours show the thresholds for percentage reductions in both newly acquired HIV infections and AIDS-related deaths by 2020 compared to 2014 levels. The ‘no increase’ contour is the threshold for satisfying the national targets (and is hence the border for the light grey region), whilst the ‘50% decrease’ contour satisfies the ambitious targets (and is hence the border for the dark grey region). In each simulation, the proportion of the budget dedicated to direct programs is optimally distributed across programs to minimize incidence, minimize deaths, and virtually eliminate MTCT.

With no additional annual spending, Kazakhstan can achieve their national targets if either a 44% [36%-58%] reduction in management costs or a 35% [30%-39%] decrease in ART unit costs can be secured, and by also optimally allocating available resources. Alternatively, the same targets could be achieved by combining smaller implementation efficiency gains and ART cost reductions whilst also allocating funds optimally (Fig 2A). For example, optimal allocative efficiency with a 20% decrease in the future cost of ART combined with a 21% [15%-29%] reduction in management costs is estimated to be sufficient to achieve the national targets. The light grey area of Fig 2A illustrates the combination of efficiency gains that are estimated to lead to Kazakhstan achieving their national targets should 2014 spending levels remain available. With no additional annual resources, Kazakhstan could further fully achieve the ambitious targets by: (1) securing at least a 51% [49%-56%] reduction in ART costs, (2) increasing the proportion of the total budget allocated to direct programs by reducing management costs, and (3) optimally allocating available resources across treatment and prevention programs. The dark grey area of Fig 2A illustrates the corresponding combination of efficiency gains estimated to achieve the ambitious targets.

Should the total annual HIV budget be reduced by 20% due to international donor withdrawal without replacement from domestic sources, our results indicate a 35% decrease in ART costs would need to be combined with a 32% [26%-36%] reduction in management costs and optimal allocative efficiency for national targets to be realized (Fig 2B). Further, if substantial implementation efficiency gains (at least 24% [19%-31%] reductions in management costs), substantial technical efficiency gains (at least 70% [64%-74%] reductions in future ART unit costs), and also optimal allocative efficiency are attained, we estimate Kazakhstan could achieve the ambitious targets without replenishing funds currently sourced from international donors (Fig 2B), i.e. with only 80% of the 2014 HIV budget applied through to 2020.

**Fig 3:**
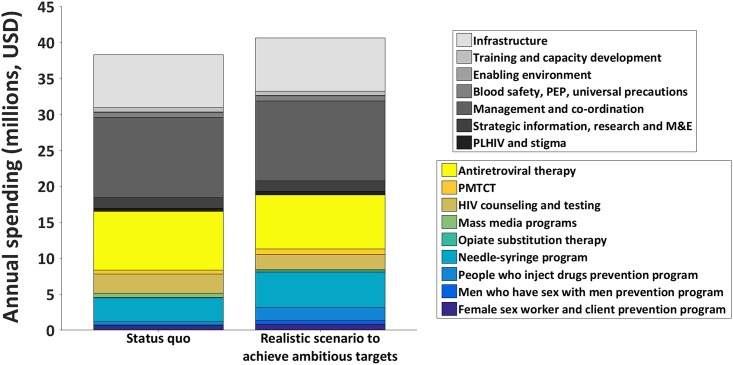
Allocations to programs under the status-quo scenario and the realistic scenario to achieve ambitious targets. This figure illustrates the 2014 allocation to HIV programs in Kazakhstan, alongside the optimal distribution of funds under the realistic scenario to achieve ambitious targets. This bar illustrates the ‘best-fit’ result, whilst uncertainty bounds around the program allocations are presented in Table 1 and S4 Fig.

Of the array of results that achieved the ambitious targets, the scenario of attaining a 67% reduction in the cost of ART along with a 19% [14%-27%] reduction in management costs (with the current national budget distributed optimally across programs) was identified as being realistic by country representatives and key stakeholders. Henceforth, we refer to this scenario as the ‘realistic scenario to achieve ambitious targets’. The optimally allocated distribution of funds under this realistic scenario is illustrated in Fig 3 alongside the 2014 allocations of the status-quo scenario. Uncertainty bounds around the optimal allocation of funds in the realistic scenario to achieve ambitious targets are presented Table 1 and S4 Fig.

**Table 1:** Allocations to programs, associated coverages levels, and key epidemiological outcomes. This table summarizes the allocation to–and associated coverage of–each modeled program for the status-quo scenario and also the multi-efficiency scenario to achieve ambitious targets. This table also contains several key summary epidemiological outcomes from the modeled scenarios. We note here that whilst total 2015 spending is constrained by the relevant assumption of available budget, spending in consecutive years may vary slightly due to treatment liabilities, where treatment *coverage* is held constant rather than the *number of people* receiving treatment.

Analysis to end of 2020	Status-quo	Realistic scenario to achieve ambitious targets
Allocation to female sex worker & client prevention program in 2015	$604,449	$848,673 [$737,336 - $1,028,342]
Allocation to men who have sex with men prevention program in 2015	$128,140	$555,350 [$376,739 - $622,025]
Allocation to people who inject drugs program in 2015	$456,213	$1,746,664 [$1,144,604 - $2,004,516]
Allocation to needle-syringe program in 2015	$3,307,210	$4,944,293 [$4,103,641 - $5,066,733]
Allocation to opiate substitution therapy in 2015	$73,775	$157,946 [$73,775 - $495,909]
Allocation to mass media programs in 2015	$591,654	$211,835 [$0 - $545,436]
Allocation to HIV counselling and testing in 2015	$2,647,059	$2,082,904 [$2,253,772 - $3,303,774]
Allocation to PMTCT in 2015	$551,634	$753,810 [$710,264 - $862,413]
Allocation to antiretroviral therapy in 2015	$8,136,601	$7,504,780 [$7,179,095 - $7,882,563]
Total HIV spending 2015	$38,287,715	$38,287,715
Total direct program spending 2015–2020	$101,133,224 [$101,000,724 - $101,288,174]	$110,510,510 [$107,737,855 - $116,750,931]
Total indirect program spending 2015–2020	$130,746,000	$116,888,759 [$110,949,942 - $119,858,168]
Total HIV spending 2015–2020	$231,879,224 [$231,746,724 - $232,034,174]	$227,399,269 [$226,145,827 - $228,746,556]
FSW & client condom program coverage	78%	90% [85% - 94%]
MSM condom program coverage	8%	19% [17% - 19%]
PWID condom program coverage	19%	51% [41% - 54%]
Needle-syringe program coverage	51%	57% [55% - 58%]
Opiate substitution therapy program coverage	0.2%	0.8% [0% - 1%]
Mass media programs program coverage	14%	6% [0% - 13%]
People living with HIV who know their status	82% [81% - 82%]	90% [89% - 92%]
PMTCT program coverage	75%	86% [84% - 90%]
Antiretroviral therapy coverage (eligibility: diagnosed and CD4 cell count<500 cells/mm^3^)	47% [46% - 49%]	99% [97% - 99%]
Those on treatment who are virally suppressed	87% [85% - 89%]	87% [85% - 89%]
Number on 1st-line treatment	5,129 [5,094–5,169]	14,667 [14,400–15,374]
Number on 2nd-line treatment	551 [550–552]	1,057 [1,37–1,096]
Number eligible for treatment (eligibility: diagnosed and CD4 cell count<500 cells/mm^3^)	11,983 [11,594–12,377]	15,942 [15,711–16,687]
Cumulative new infections 2015–2020	9,471 [8,896–10,065]	3,971 [3,920–4,295]
Cumulative AIDS-related deaths 2015–2020	6,505 [6,185–6,819]	2,287 [2,200–2,324]
Overall prevalence in 2020	0.14% [0.14% - 0.15%]	0.13% [0.13% - 0.14%]
Number of people living with HIV in 2020	19,171 [18,360–19,998]	17,889 [17,530–18,510]
New infections averted by 2020	Baseline	5,500 [5,052–5,832]
AIDS-related deaths averted by 2020	Baseline	4,218 [4,48–4,466]

In the realistic scenario that achieves the ambitious targets, more funds were directed to programs for key populations than currently allocated: coverage for female sex worker and client prevention programs increased from 78% to 90% [85%-94%], prevention programs for men who have sex with men from 8% to 19% [17%-19%], programs for people who inject drugs from 19% to 51% [41%-54%], and needle-syringe programs from 51% to 57% [55%-58%]. Program coverage also increased for OST (from 0.2% to 0.8% [0.2%-1.1%]) and PMTCT programs (from 75% to 86% [84%-90%]), although coverage of mass media programs decreased from 14% to 6% [0%-13%]. ART coverage (where those diagnosed with a CD4 count of <500 cells per cubic millimeter of blood are eligible [30]) increased from 47% in the status-quo scenario to >90% under the realistic scenario to achieve ambitious targets. Due to the assumed decrease in the cost of ART procurement in this scenario, the annual cost of such a program is less ($7.5 million in 2015) than for the status-quo scenario ($8.1 million in 2015). In addition, with a 19% [14%-27%] reduction in management costs obtained through implementation efficiencies, the percentage of total HIV budget consumed by indirect costs from 2015–2020 is reduced from 56% to 51% [49%-53%] under the realistic scenario to achieve ambitious targets compared to the status-quo scenario. A summary of these results is presented in Table 1.

**Fig 4:**
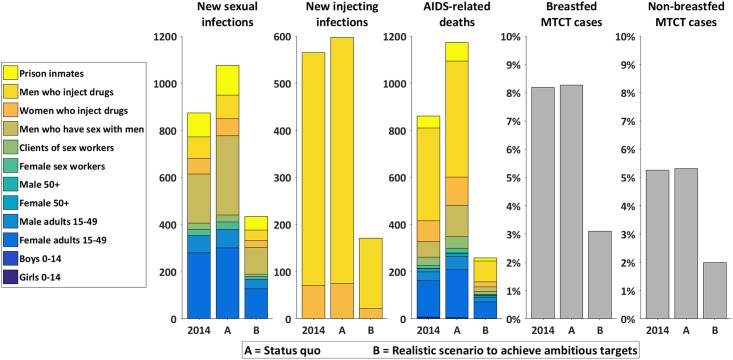
Epidemiological outcomes under the status-quo scenario and realistic scenario to achieve ambitious targets in 2020 compared to 2014. This figure illustrates the corresponding epidemiological outcomes that are estimated to arise by implementing the allocations presented in Fig 3 (status-quo scenario, and the optimized realistic scenario to achieve ambitious targets) between 2015 and 2020. In each sub chart, the first column represents the value of the indicator in 2014.

Our results indicate that annual newly-acquired sexual- and injecting-related HIV infections and AIDS-related deaths will increase in 2020 from 2014 levels under the baseline status-quo (Fig 4). In contrast, annual newly-acquired sexual- and injecting-related infections are projected to decrease by 50% and 63%, respectively, under the realistic scenario of achieving ambitious targets. AIDS-related deaths are estimated to decrease by 71% under this scenario. Virtual elimination of mother-to-child-transmission would also be achieved in the ambitious targets scenario, which was not the case when status-quo spending was projected. This translates to an estimated 5,500 newly-acquired HIV infections and 4,220 AIDS-related deaths being averted under the realistic scenario to achieve ambitious targets compared to the status-quo scenario between 2015 and 2020. Due to the epidemiological impact of the realistic scenario to achieve ambitious targets, future treatment liabilities decrease and, over time, the cost of the HIV response begins to decrease accordingly; so much so that between 2015 and 2020 the total cumulative cost of the ambitious targets scenario is estimated to be $4.5 million less ($227.4 million) than the status-quo scenario ($231.9 million).

## Discussion

The Government of Kazakhstan has moved to increase domestic responsibility of the HIV budget over recent years; between 2007–2011 the domestic share of ART funding increased from 7% to 100% [6]. Many other countries have already implemented, or are soon likely to require, a similar transition from international to domestic sources to finance their national HIV responses [31]. Historically, however, there has often been delays between external funding withdrawal and domestic funding scale up [32]. Amongst other factors, this may be due to withstanding political sensitivities surrounding key-affected populations such as people who inject drugs, female sex workers, and men who have sex with men [32]. In this context of domestic responsibility for key-affected population programs, a recent success story has emerged from Croatia. Following large scale external support between 2003–2006, primarily from GFATM, Croatia has since transitioned to a fully domestically-financed response, and has further scaled up key programs [32]. A key factor in Croatia’s successful transition to domestic financing was the cooperation between the government and non-government organizations to target resources to otherwise marginalized populations [32]. Kazakhstan itself has also achieved some success in transitioning funding for key-affected populations.

Our modeling results indicate that by continuing the current HIV intervention strategy Kazakhstan will likely have more annual newly-acquired infections and AIDS-related deaths in 2020 than 2014. In the absence of allocative, implementation, and technical efficiencies, we estimate that a 32% increase in total HIV budget would be required to achieve national targets. Alternatively, our modeling findings suggest the same targets could be satisfied with no increase in budget by reallocating funding to focus on programs for key-affected populations, reducing management costs, reducing ART procurement costs and consequently increasing treatment coverage. More ambitious epidemiological targets could also be attained without additional overall resourcing. Our results suggest that Kazakhstan could potentially reduce new HIV-infections and AIDS-related deaths by 50% of 2014 levels by 2020 even without replenishment of 20% of the HIV budget expected to be withdrawn. However, such an outcome would require substantial implementation efficiency gains and very large reductions in ART costs beyond the levels deemed reasonable by country representatives and key stakeholders.

Allocative efficiency studies have been carried out in many countries to highlight potential inefficiencies in current investment approaches [33–39]. A recent study of the concentrated epidemic in Sudan concluded that reallocating resources from un-targeted general population programs to treatment programs, prevention programs of female sex workers, and prevention programs for men who have sex with men could result in substantial epidemiological gains [25]. In light of these findings, the Sudanese government has since shifted funding priorities to closer align with the findings of the study [40]. Within the EECA region, Armenia has undertaken several studies to assess the allocative efficiency of their HIV response [41, 42], and have used the evidence to advocate for resources to be targeted towards cost-effective programs for key-affected populations such as short-term labour migrants [43].

As with all mathematical modeling studies, it is important that our findings be interpreted with an appreciation of the inherent assumptions. We assumed that it is possible for ART costs to be reduced by up to 80%. Whether such reductions in treatment costs are plausible in Kazakhstan is debatable. A 2012 analysis found that implementing partners in Mozambique were able to reduce the mean unit cost of ART by 45% between 2009 and 2011 by adjusting inefficient service delivery models [44]. Along with more efficient drug procurement via the UN and GFATM systems, several other factors may synergistically contribute to reduce future costs of ART in Kazakhstan. Tenofovir Disoproxil Fumarate (TDF)–a nucleotide reverse transcriptase inhibitor commonly used in fixed-dose combination ART–is due to expire in 2017 [45]. Typically, both originator and generic drug prices decrease as associated patents near expiration [9], hence it is likely that the cost of purchasing current combinations of ART will decrease in the near future. Further, trials have shown that by optimizing antiretroviral dosages such that any negative effect on viral suppression is negligible can help to cut the cost of HIV treatment. A number of dose optimization trials which compared the virological efficacy of a reduced dose versus standard dose of an antiretroviral drug have recently been completed [46, 47], finding that several antiretroviral agents show no difference in virological suppression rates across the different dosages. The implication of reduced antiretroviral dosages on the cost of treatment programs could potentially be substantial.

Our analyses consider efficiency gains in both direct and indirect programs. As an indirect cost that absorbs a large portion of national funding that cannot be mathematically optimized due to its unquantifiable effect on epidemiological outcomes, we assume that management costs can be reduced whilst still allowing for essential functions to be maintained. A 2012 study of the HIV response in Ukraine estimated that costs associated with program staff could be reduced on average by 18% whilst maintaining the same level of outputs [48]. In this paper, we focus on a scenario that incorporates a ~20% reduction of management costs; a selection that was made in line with national expert opinion as it was deemed to be realistically attainable. We further consider scenarios that assume management costs are able to be reduced by up to 50% of 2014 levels without detrimental effect on the direct treatment and prevention programs that such spending supports. Such results are included to indicate what epidemiological gains may be attainable if such efficiency gains can be achieved. However, additional setting-specific work is needed to focus on specific implementation efficiency analyses to understand the plausibility and potential impact of such spending cuts. Further, our results assume that achieving the instant scale up and scale down of treatment and prevention programs to optimal levels is plausible. In reality, this may not be the case as certain logistical, ethical, and political constraints may exist that slow or even prevent the scale up of programs to optimal levels.

Our modeling findings suggest that Kazakhstan will not be able to substantially reduce new infections and deaths unless coverages of key prevention and treatment services are scaled up. Achieving the proposed high coverage of effective ART will require extensive demand generation and adherence support. Among people who inject drugs, access to OST has been shown to substantially improve adherence to antiretroviral therapy [49, 50], and is thus likely to be an essential condition for a successful treatment program amongst this risk group. Additional outreach support programs may also be required to increase access and adherence to ART for key population groups such as female sex workers, men who have sex with men, and people who inject drugs.

Faced with impending donor withdrawal and a non-decreasing HIV epidemic, it will become increasingly important for Kazakhstan’s HIV response to achieve more with limited available resources. This analysis can help to guide policy makers in directing available resources to achieve the greatest yield from HIV investments, and ultimately turn the tide of the national epidemic.

## Supporting information

S1 Fig Calibration of Optima to the HIV epidemic in Kazakhstan. Using all available demographic, epidemiological, behavioral, and clinical data we calibrated Optima to the HIV epidemic in Kazakhstan between 2000 and 2014. This was achieved by varying input parameters within their uncertainty bounds such that model projections were within the uncertainty bounds of empirical data on population group prevalence, number of new diagnoses per year, and the number of people on treatment. On inspection, the Optima outputs of HIV prevalence, number of people living with HIV, and number of AIDS-related deaths were found to be similar in magnitude and trend to estimates produced by the Spectrum model. Generally, Optima closely matches the available HIV prevalence and treatment data. The early female sex worker prevalence data was deemed to be less reliable than post-2006 data by the Kazakhstan country team, and the apparent downward trend of female sex worker prevalence was not believed to be accurate. As such, we favored the more recent female sex worker prevalence data and the clients of sex worker prevalence data during the calibration process. Dark grey discs represent available data for HIV prevalence. The dark grey lines attached to these discs represent uncertainty bounds. The solid pink curve is the best fitting simulation, and the shaded pink region shows the range of the uncertainty simulations.
(DOCX)

S2 Fig Calibration of model to ART scale-up data in Kazakhstan. Black discs represent available data for the number of people on first and subsequent lines of anti-retroviral treatment. The solid pink curve is the best fitting simulation and the shaded pink region represents the range of uncertainty simulations.
(DOCX)

S3 Fig Relationships between program costs and associated programmatic outcomes for the range of programs and target populations modelled. These curves are the result of combining the intermediate cost-coverage and coverage-outcome relationships for each programmatic outcome. These relationships were defined during a workshop held in November 2014 in Yerevan, Armenia, which was co-hosted by the Global Fund to Fight AIDS, Tuberculosis and Malaria, UNAIDS, the United Nations Development Program, the World Bank, and various other partners. This process was conducted by the Optima HIV modelling team in consultation with Kazakhstan country representatives and other stake holders. In several cases, the magnitude of the programmatic outcome data was not trusted by the Kazakhstan country team, and adjustments to the curves were made accordingly. The Kazakhstan country representatives approved the set of curves presented here during the workshop. Black discs represent available program spending verses program-related outcome data. The solid curve is the best estimate cost-outcome curve, and the shaded region represents the range of uncertainty considered in the each of the cost-outcome relationships.
(DOCX)

S4 Fig Uncertainty around optimal allocations. An uncertainty analysis was undertaken to determine how uncertainties in both model calibration and cost-outcome relationships impacted on allocation recommendations. Forty baseline model simulations were sampled from an ensemble of projections within the uncertainty bounds of the model calibration, as were 40 samples of each of the cost-outcome relations within their respective uncertainty bounds. Here we present the impact of such uncertainty on optimal allocations and associated coverages under different assumptions of future ART cost reduction. In each case, the ambitious targets defined in the manuscript are fully achieved. The blue dots represent the optimal allocations obtained from the analysis of the ‘best-estimate’ model calibration and cost-outcome curves. The blue shaded regions represent the range of uncertainty in the optimal allocations. The red dots and red shaded region represent the best-estimate and uncertainty optimal program coverages associated with the optimal program allocations. Subfigure A shows the impact of uncertainty on optimal allocations and associated coverages when future ART costs are reduced by 67% (a 3-fold reduction). In subfigure B future ART costs are fixed at 2014 levels, and in subfigure C future ART are reduced by 80% (a 5-fold reduction). The program specific coverages corresponding to optimal spending patterns are very similar in each of these cases. This phenomenon, along with the consistently tight uncertainty bounds around the best-fit optimal result, suggests that these optimal coverage recommendations for achieving the ambitious targets are a robust finding.
(DOCX)
