# Supplementary material for: Kazakhstan can achieve ambitious HIV targets despite expected donor withdrawal by combining improved ART procurement mechanisms with allocative and implementation efficiencies
Source: PLoS One. 2017 Feb 16;12(2):e0169530. doi: 10.1371/journal.pone.0169530 (PMC5313190; doi:10.1371/journal.pone.0169530)

**S1 Fig. Calibration of Optima to the HIV epidemic in Kazakhstan.** Using all available demographic, epidemiological, behavioral, and clinical data we calibrated Optima to the HIV epidemic in Kazakhstan between 2000 and 2014. This was achieved by varying input parameters within their uncertainty bounds such that model projections were within the uncertainty bounds of empirical data on population group prevalence, number of new diagnoses per year, and the number of people on treatment. On inspection, the Optima outputs of HIV prevalence, number of people living with HIV, and number of AIDS-related deaths were found to be similar in magnitude and trend to estimates produced by the Spectrum model. Generally, Optima closely matches the available HIV prevalence and treatment data. The early female sex worker prevalence data was deemed to be less reliable than post-2006 data by the Kazakhstan country team, and the apparent downward trend of female sex worker prevalence was not believed to be accurate. As such, we favored the more recent female sex worker prevalence data and the clients of sex worker prevalence data during the calibration process. Dark grey discs represent available data for HIV prevalence. The dark grey lines attached to these discs represent uncertainty bounds. The solid pink curve is the best fitting simulation, and the shaded pink region shows the range of the uncertainty simulations.


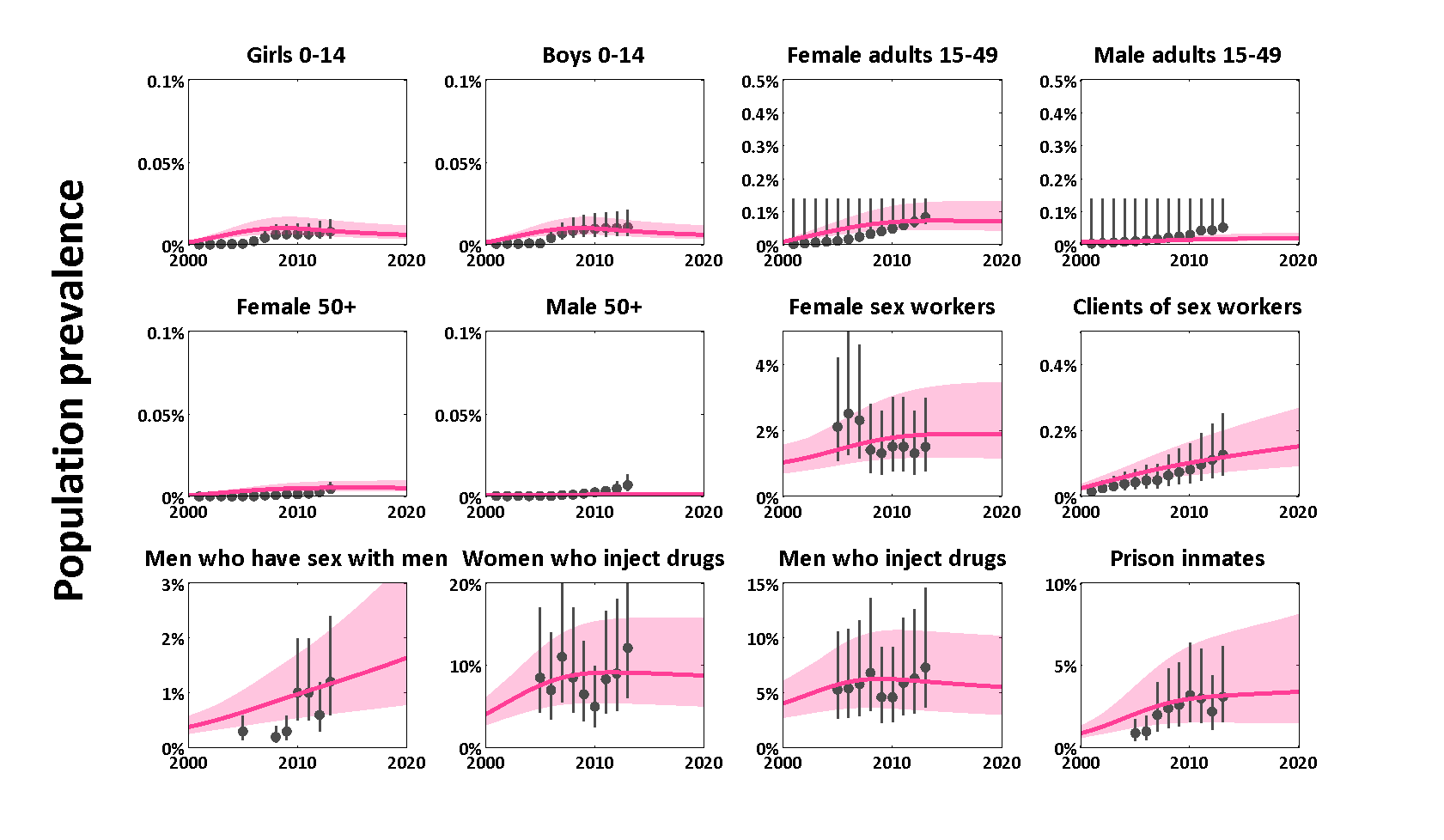

Supplement: S1 Fig — Using all available demographic, epidemiological, behavioral, and clinical data we calibrated Optima to the HIV epidemic in Kazakhstan between 2000 and 2014. This was achieved by varying input parameters within their uncertainty bounds such that model projections were within the uncertainty bounds of empirical data on population group prevalence, number of new diagnoses per year, and the number of people on treatment. On inspection, the Optima outputs of HIV prevalence, number of people living with HIV, and number of AIDS-related deaths were found to be similar in magnitude and trend to estimates produced by the Spectrum model. Generally, Optima closely matches the available HIV prevalence and treatment data. The early female sex worker prevalence data was deemed to be less reliable than post-2006 data by the Kazakhstan country team, and the apparent downward trend of female sex worker prevalence was not believed to be accurate. As such, we favored the more recent female sex worker prevalence data and the clients of sex worker prevalence data during the calibration process. Dark grey discs represent available data for HIV prevalence. The dark grey lines attached to these discs represent uncertainty bounds. The solid pink curve is the best fitting simulation, and the shaded pink region shows the range of the uncertainty simulations. (DOCX) [file pone.0169530.s001.docx]
