# Supplementary material for: Kazakhstan can achieve ambitious HIV targets despite expected donor withdrawal by combining improved ART procurement mechanisms with allocative and implementation efficiencies
Source: PLoS One. 2017 Feb 16;12(2):e0169530. doi: 10.1371/journal.pone.0169530 (PMC5313190; doi:10.1371/journal.pone.0169530)

**S2 Fig.** **Calibration of model to ART scale-up data in Kazakhstan.** Black discs represent available data for the number of people on first and subsequent lines of anti-retroviral treatment. The solid pink curve is the best fitting simulation and the shaded pink region represents the range of uncertainty simulations.


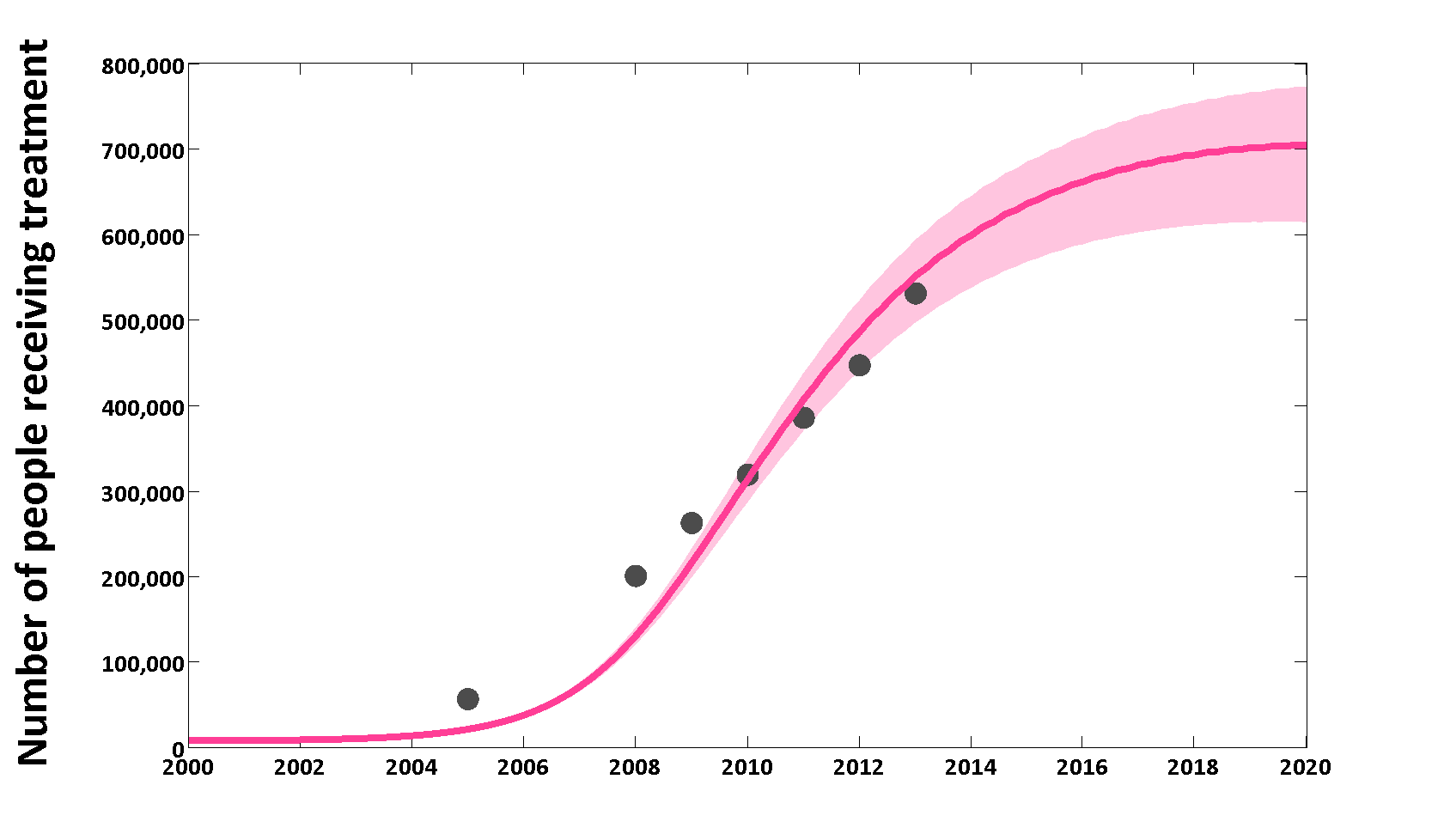

Supplement: S2 Fig — Black discs represent available data for the number of people on first and subsequent lines of anti-retroviral treatment. The solid pink curve is the best fitting simulation and the shaded pink region represents the range of uncertainty simulations. (DOCX) [file pone.0169530.s002.docx]
