# Supplementary material for: Kazakhstan can achieve ambitious HIV targets despite expected donor withdrawal by combining improved ART procurement mechanisms with allocative and implementation efficiencies
Source: PLoS One. 2017 Feb 16;12(2):e0169530. doi: 10.1371/journal.pone.0169530 (PMC5313190; doi:10.1371/journal.pone.0169530)

**S3 Fig. Relationships between program costs and associated programmatic outcomes for the range of programs and target populations modelled.** These curves are the result of combining the intermediate cost-coverage and coverage-outcome relationships for each programmatic outcome. These relationships were defined during a workshop held in November 2014 in Yerevan, Armenia, which was co-hosted by the Global Fund to Fight AIDS, Tuberculosis and Malaria, UNAIDS, the United Nations Development Program, the World Bank, and various other partners. This process was conducted by the Optima HIV modelling team in consultation with Kazakhstan country representatives and other stake holders. In several cases, the magnitude of the programmatic outcome data was not trusted by the Kazakhstan country team, and adjustments to the curves were made accordingly. The Kazakhstan country representatives approved the set of curves presented here during the workshop. Black discs represent available program spending verses program-related outcome data. The solid curve is the best estimate cost-outcome curve, and the shaded region represents the range of uncertainty considered in the each of the cost-outcome relationships.


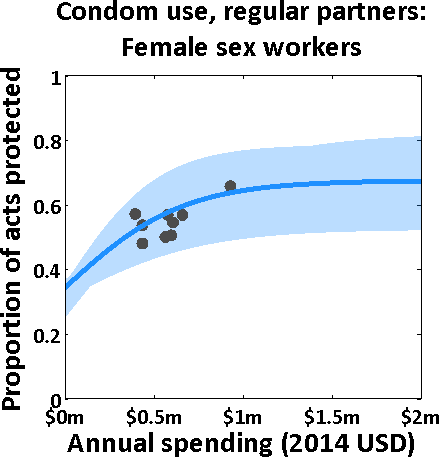

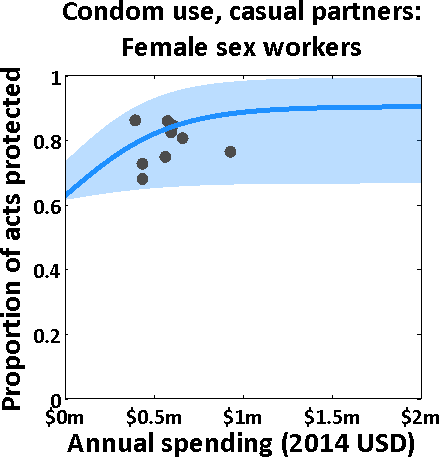

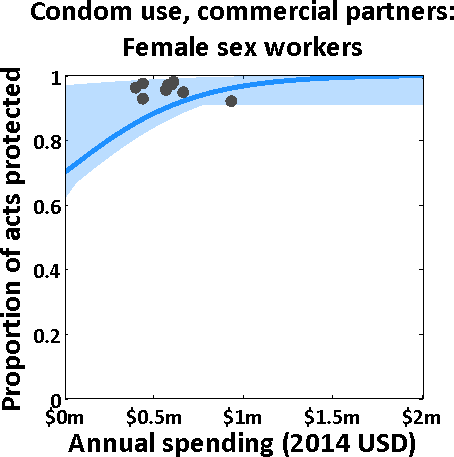


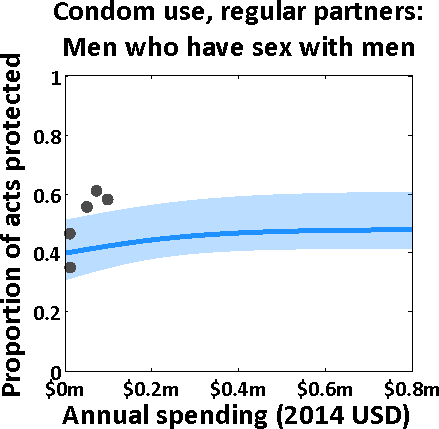

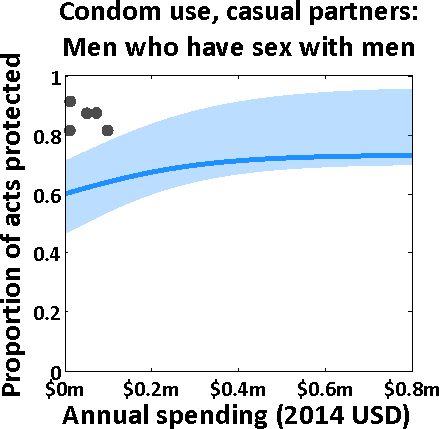

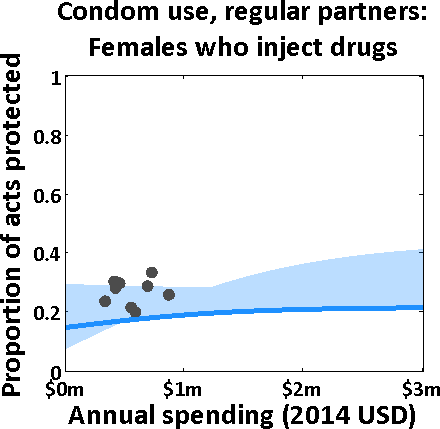


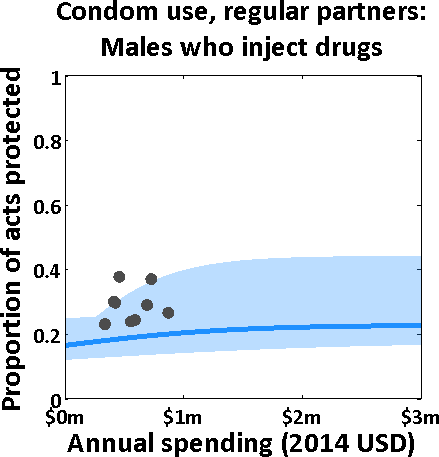

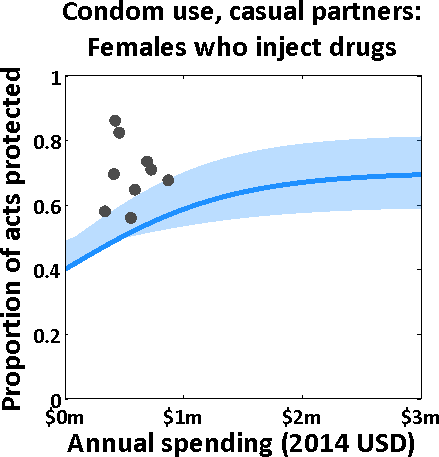

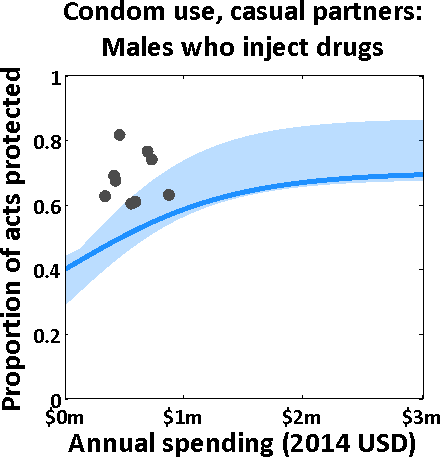


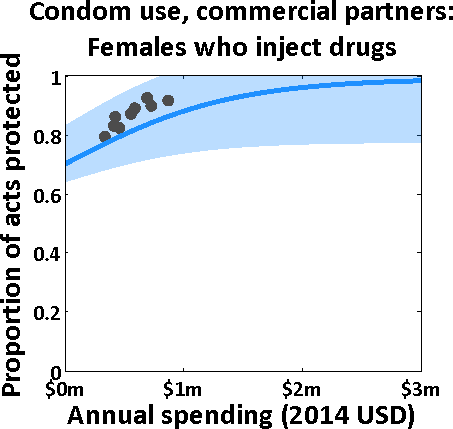

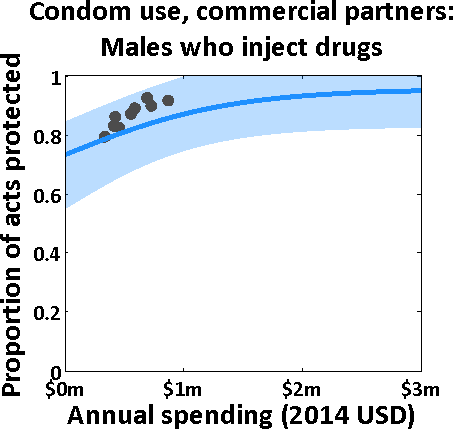

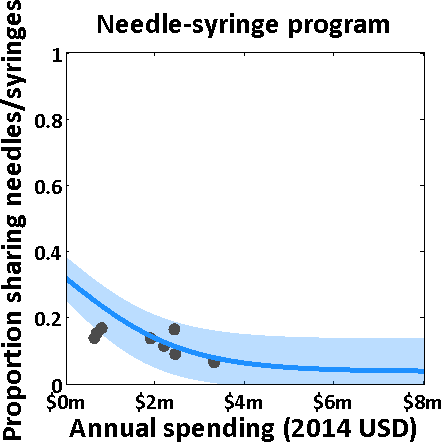


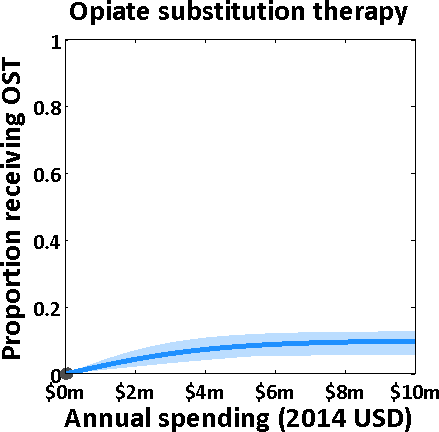

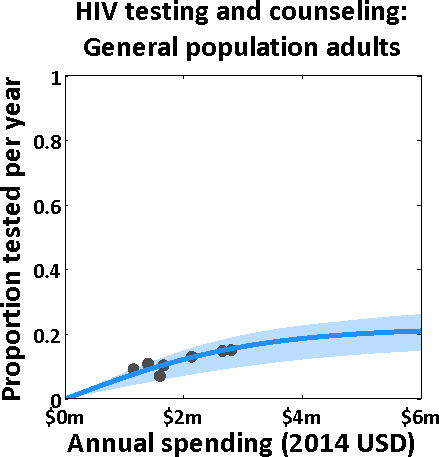

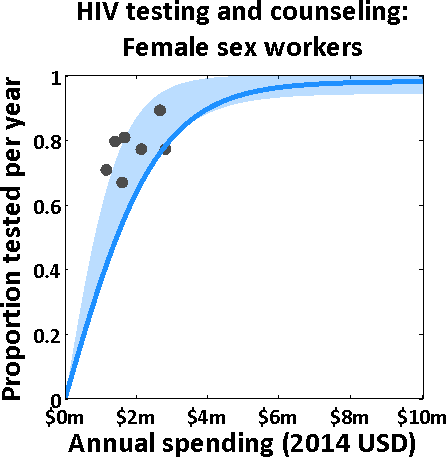


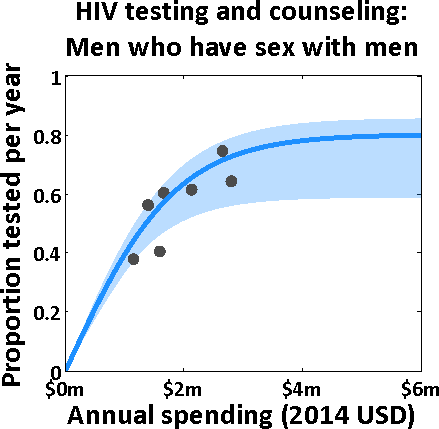

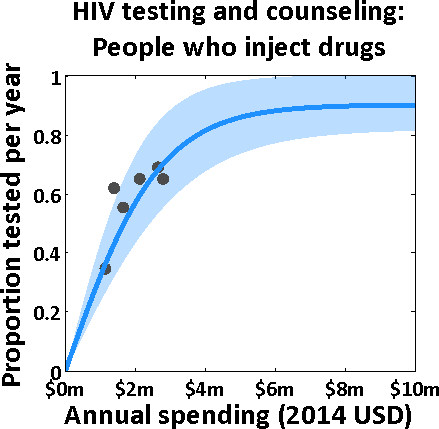

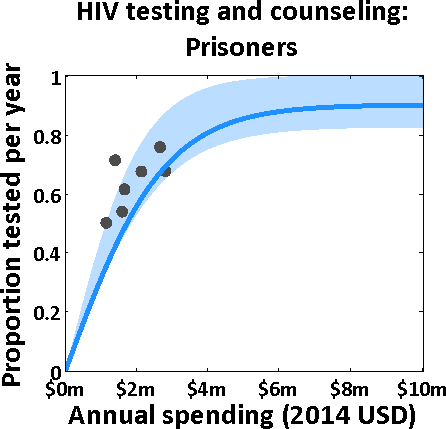


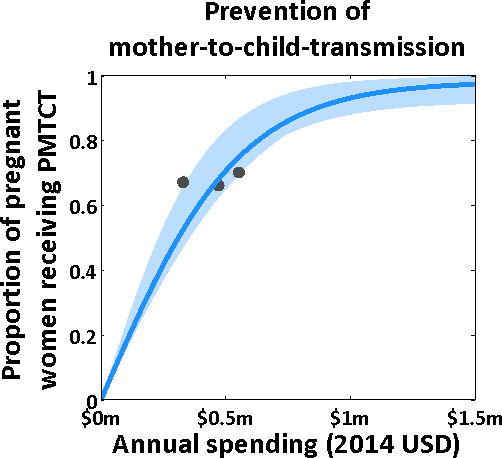

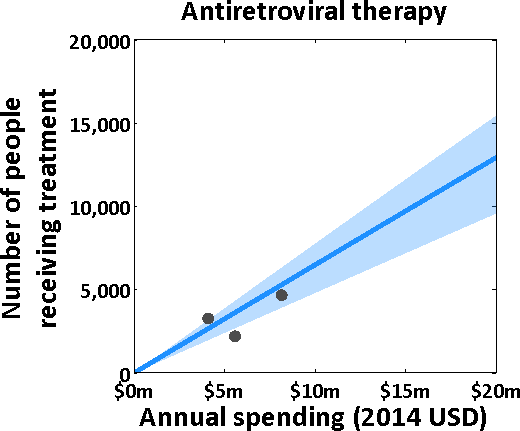

Supplement: S3 Fig — These curves are the result of combining the intermediate cost-coverage and coverage-outcome relationships for each programmatic outcome. These relationships were defined during a workshop held in November 2014 in Yerevan, Armenia, which was co-hosted by the Global Fund to Fight AIDS, Tuberculosis and Malaria, UNAIDS, the United Nations Development Program, the World Bank, and various other partners. This process was conducted by the Optima HIV modelling team in consultation with Kazakhstan country representatives and other stake holders. In several cases, the magnitude of the programmatic outcome data was not trusted by the Kazakhstan country team, and adjustments to the curves were made accordingly. The Kazakhstan country representatives approved the set of curves presented here during the workshop. Black discs represent available program spending verses program-related outcome data. The solid curve is the best estimate cost-outcome curve, and the shaded region represents the range of uncertainty considered in the each of the cost-outcome relationships. (DOCX) [file pone.0169530.s003.docx]
