# Supplementary material for: Kazakhstan can achieve ambitious HIV targets despite expected donor withdrawal by combining improved ART procurement mechanisms with allocative and implementation efficiencies
Source: PLoS One. 2017 Feb 16;12(2):e0169530. doi: 10.1371/journal.pone.0169530 (PMC5313190; doi:10.1371/journal.pone.0169530)
Supplement: S4 Fig — An uncertainty analysis was undertaken to determine how uncertainties in both model calibration and cost-outcome relationships impacted on allocation recommendations. Forty baseline model simulations were sampled from an ensemble of projections within the uncertainty bounds of the model calibration, as were 40 samples of each of the cost-outcome relations within their respective uncertainty bounds. Here we present the impact of such uncertainty on optimal allocations and associated coverages under different assumptions of future ART cost reduction. In each case, the ambitious targets defined in the manuscript are fully achieved. The blue dots represent the optimal allocations obtained from the analysis of the ‘best-estimate’ model calibration and cost-outcome curves. The blue shaded regions represent the range of uncertainty in the optimal allocations. The red dots and red shaded region represent the best-estimate and uncertainty optimal program coverages associated with the optimal program allocations. Subfigure A shows the impact of uncertainty on optimal allocations and associated coverages when future ART costs are reduced by 67% (a 3-fold reduction). In subfigure B future ART costs are fixed at 2014 levels, and in subfigure C future ART are reduced by 80% (a 5-fold reduction). The program specific coverages corresponding to optimal spending patterns are very similar in each of these cases. This phenomenon, along with the consistently tight uncertainty bounds around the best-fit optimal result, suggests that these optimal coverage recommendations for achieving the ambitious targets are a robust finding. (DOCX) [file pone.0169530.s004.docx]

**S4 Fig. Uncertainty around optimal allocations.** An uncertainty analysis was undertaken to determine how uncertainties in both model calibration and cost-outcome relationships impacted on allocation recommendations. Forty baseline model simulations were sampled from an ensemble of projections within the uncertainty bounds of the model calibration, as were 40 samples of each of the cost-outcome relations within their respective uncertainty bounds. Here we present the impact of such uncertainty on optimal allocations and associated coverages under different assumptions of future ART cost reduction. In each case, the ambitious targets defined in the manuscript are fully achieved. The blue dots represent the optimal allocations obtained from the analysis of the ‘best-estimate’ model calibration and cost-outcome curves. The blue shaded regions represent the range of uncertainty in the optimal allocations. The red dots and red shaded region represent the best-estimate and uncertainty optimal program coverages associated with the optimal program allocations. Subfigure A shows the impact of uncertainty on optimal allocations and associated coverages when future ART costs are reduced by 67% (a 3-fold reduction). In subfigure B future ART costs are fixed at 2014 levels, and in subfigure C future ART are reduced by 80% (a 5-fold reduction). The program specific coverages corresponding to optimal spending patterns are very similar in each of these cases. This phenomenon, along with the consistently tight uncertainty bounds around the best-fit optimal result, suggests that these optimal coverage recommendations for achieving the ambitious targets are a robust finding.

**A)**

**
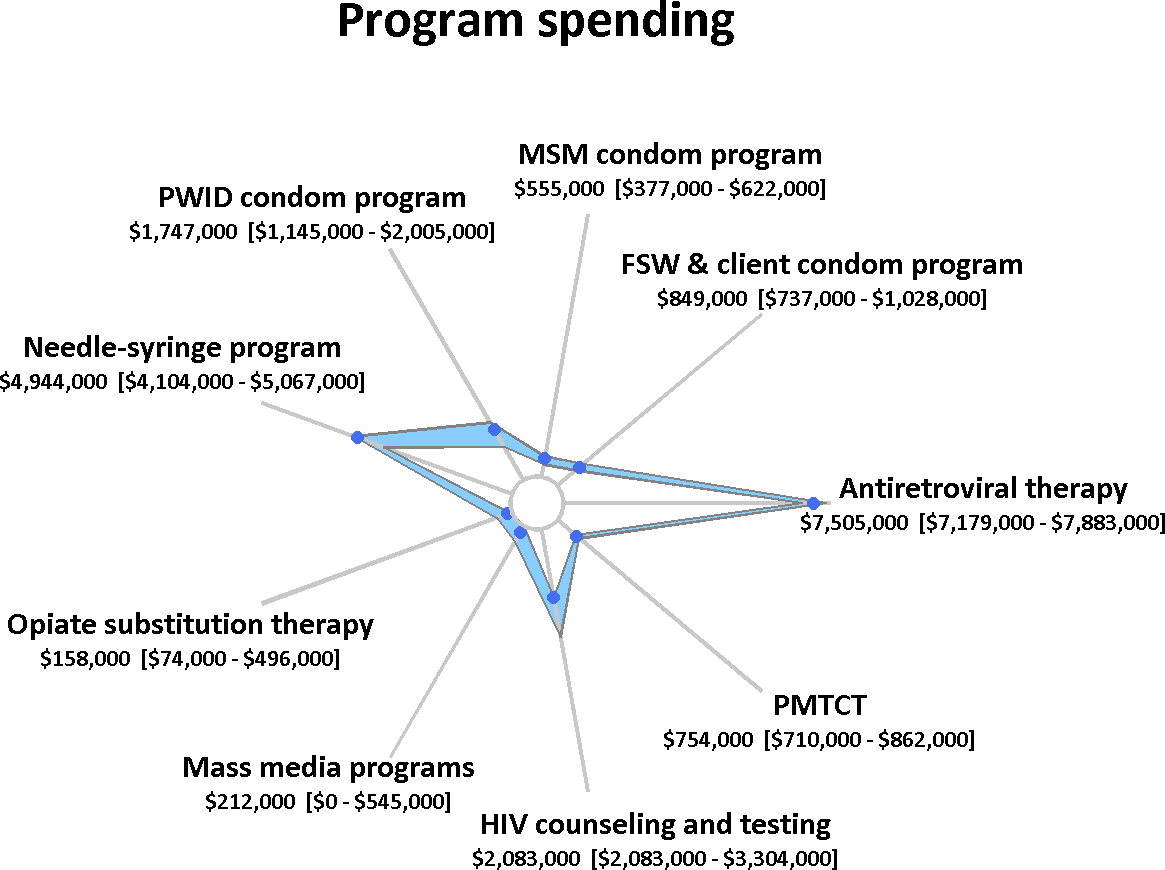

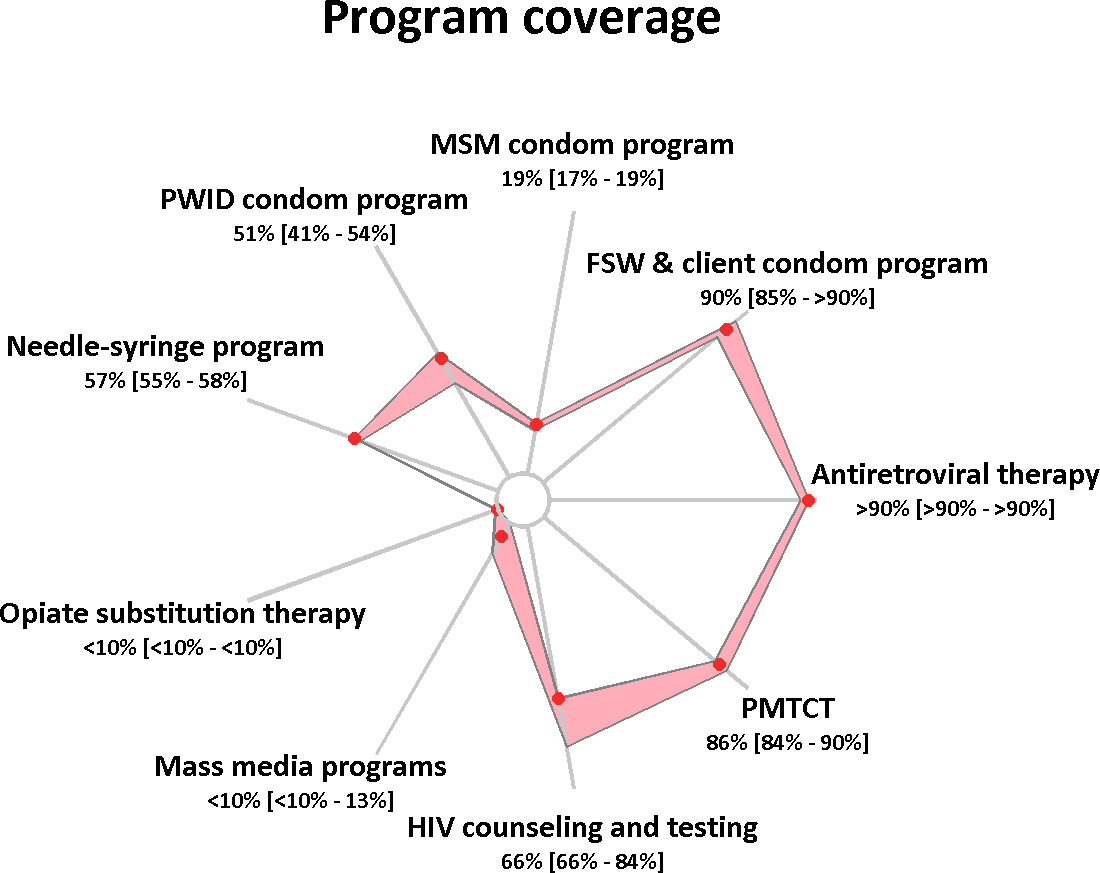
**

**B)**

**
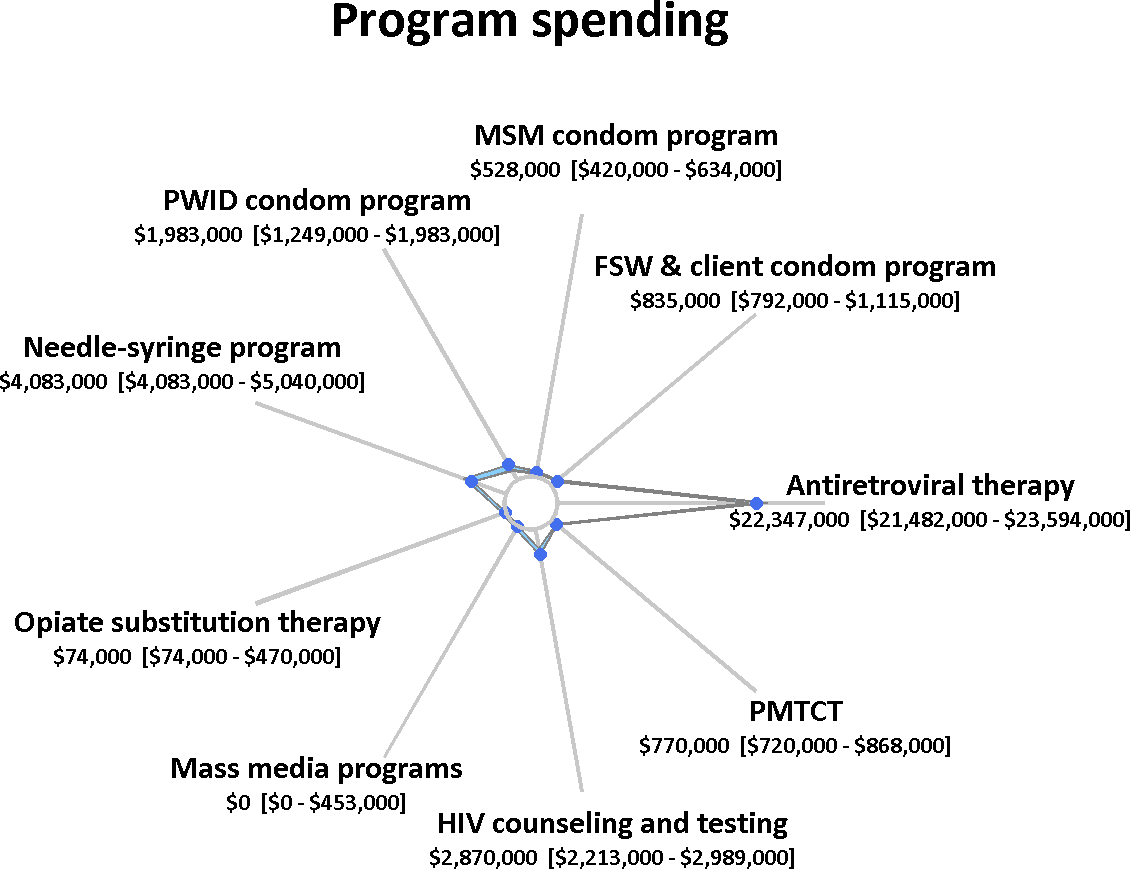

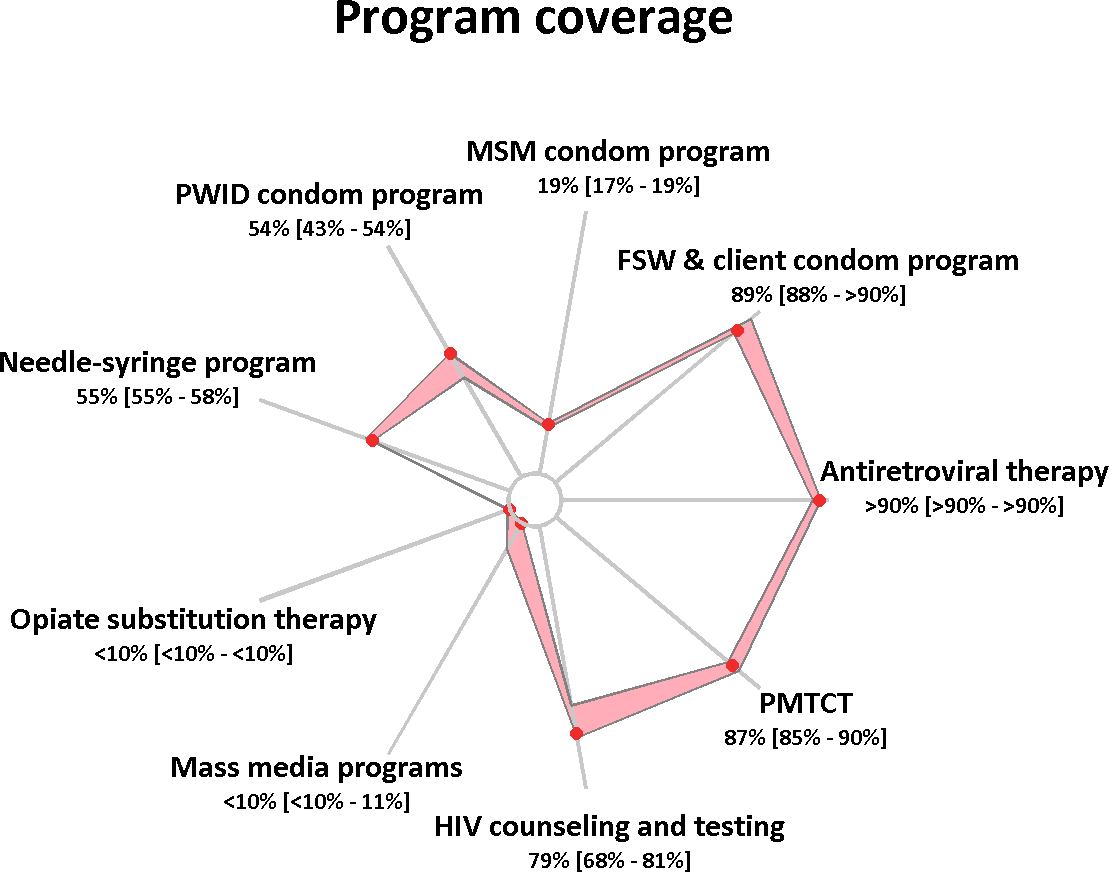
**

**C)**

**
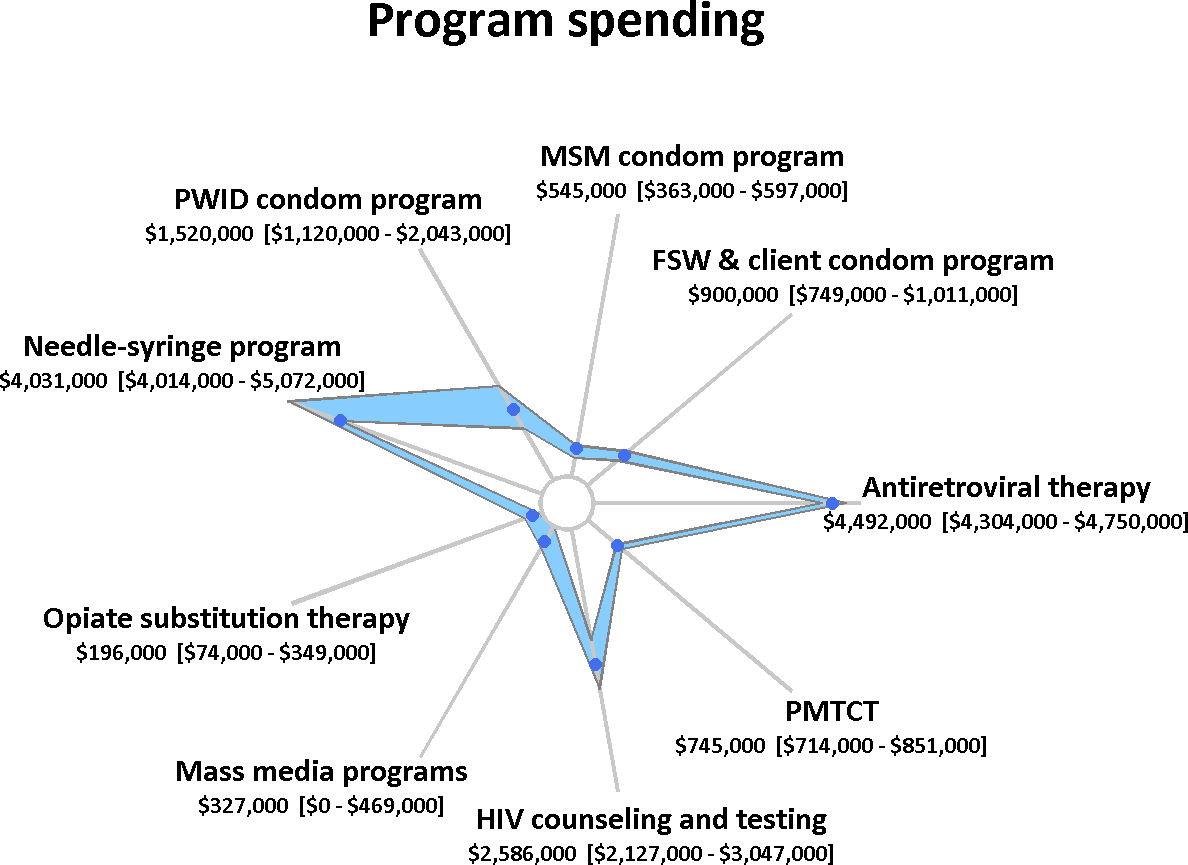

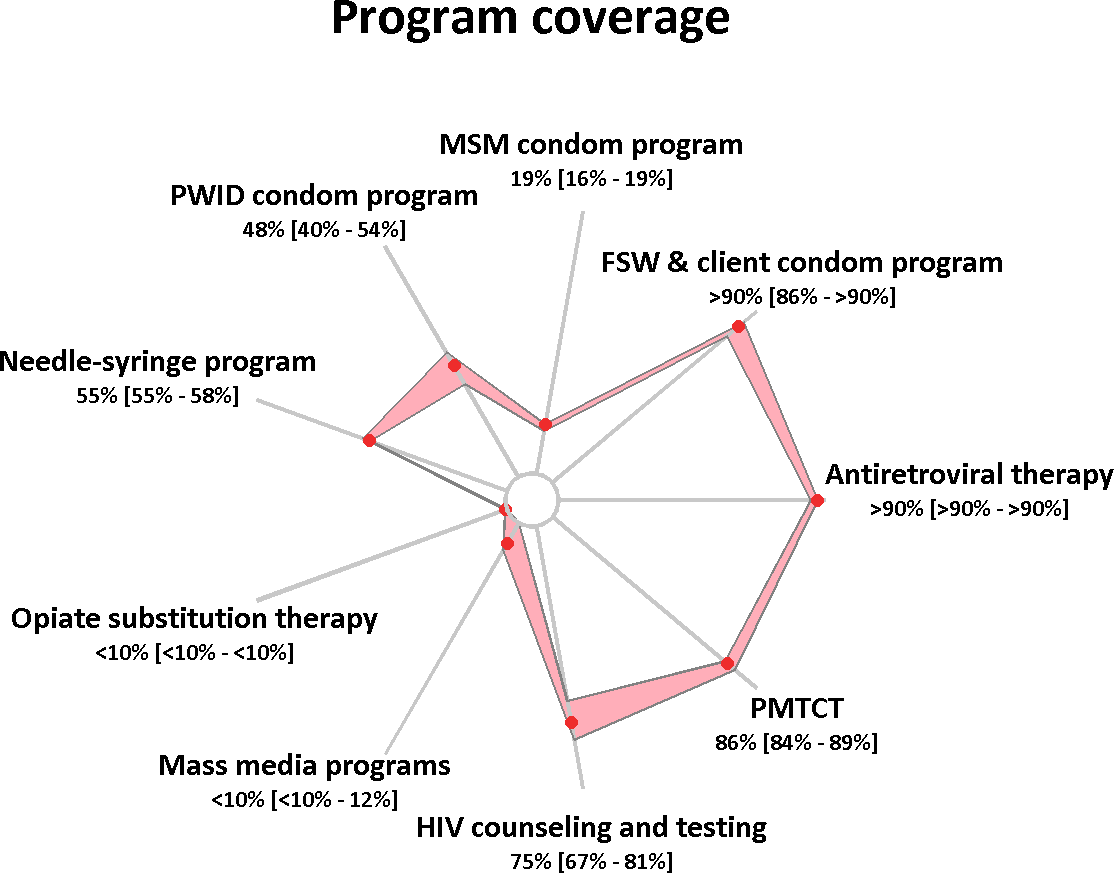
**
